# Sublobar resection versus lobectomy in solid-type, clinical stage IA, non-small cell lung cancer

**DOI:** 10.1186/1477-7819-12-215

**Published:** 2014-07-16

**Authors:** Hyun Woo Jeon, Young-Du Kim, Kyung Soo Kim, Sook Whan Sung, Hyung Joo Park, Jae Kil Park

**Affiliations:** 1 Department of Thoracic and Cardiovascular Surgery, Bucheon St. Mary’s Hospital, College of Medicine, The Catholic University of Korea, 327 Sosaro, Wonmi-gu, Bucheon, Republic of Korea; 2 Department of Thoracic and Cardiovascular Surgery, Seoul St. Mary’s Hospital, College of Medicine, The Catholic University of Korea, 222 Banpo-daero, Seocho-gu, Seoul 137-701, Republic of Korea

**Keywords:** Non-small cell lung cancer, Sublobar resection, Clinical stage IA

## Abstract

**Background:**

Recent studies have demonstrated that sublobar resection is not inferior to lobectomy for peripheral early lung cancer with ground-glass opacification. However, the effect of sublobar resection on solid-type early lung cancer is controversial. The aim of this study was to compare clinical outcomes of patients who have undergone sublobar resection or lobectomy for solid-type, early-stage, non-small cell lung cancer (NSCLC).

**Methods:**

This study was a retrospective review of the records of patients who underwent lobectomy or sublobar resection between March 2000 and September 2010 for clinical stage IA NSCL. Patients with pure ground-glass opacities or death within 30 days after surgery were excluded. Disease-free interval, survival, and prognostic factors were analyzed.

**Results:**

Thirty-one patients and 133 patients underwent sublobar resection and lobectomy, respectively. There were significant differences in age (*P* <0.001), cardiovascular disease (*P* = 0.001), and diffusing capacity of the lung for carbon monoxide (DLCO) (*P* <0.001). The patients with lobectomy had a significantly longer disease-free interval (*P* <0.001) and survival (*P* = 0.001). By multivariate analysis, sublobar resection (*P* = 0.011), lymphatic vessel invasion (*P* = 0.006), and number of positive lymph nodes (*P* = 0.028) were predictors for survival. Sublobar resection (*P* <0.001), visceral pleural invasion (*P* = 0.002), and lymphatic vessel invasion (*P* <0.001) were predictors for disease-free interval.

**Conclusions:**

Lobectomy should remain the standard surgical procedure for solid-type, clinical stage IA, NSCLC.

## Background

Lung cancer is the most common cause of cancer-related death worldwide. Surgical resection for stage IA, non-small cell lung cancer (NSCLC) is the best option for curative treatment, with a 5-year survival rate approaching 70%. Lobectomy with mediastinal lymph node dissection has been considered standard treatment. However, some early-stage patients undergo sublobar resection because of poor cardiopulmonary reserve or other medical comorbidities. Sublobar resection consists of segmentectomy or wedge resection with or without lymph node dissection.

For ground-glass opacities on imaging, which have been found to be slowly growing and noninvasive tumors [1], sublobar resection is not inferior to lobectomy. However, in solid-type or solid-dominant early lung cancer, the efficacy of sublobar resection is controversial. Several studies have revealed that sublobar resection was associated with poor outcomes and higher recurrence rates [2,3]. However, because of advances in imaging study and surgical technique, sublobar resection is a reasonable approach for patients with early lung cancers that are 2 cm or smaller by recent study [4]. We studied the clinical outcomes of patients with solid-type early lung cancers who underwent sublobar resection or lobectomy.

## Methods

### Patients

This was a retrospective review that was approved by the Institutional Review Board at the Seoul St. Mary’s Hospital (Republic of Korea). Between March 2000 and September 2010, 204 patients had clinical stage IA NSCLC, according to the 7th edition of the TNM classification for lung cancer. Patients with ground-glass opacity on imaging, a tumor with predominant ground-glass opacification, or who died within 30 days after surgery were excluded. A total of 38 patients showed ground-glass opacity or ground-glass opacity dominant lesion (GGO >50%) on chest tomography. Two patients died from pneumonia and acute respiratory distress syndrome after operation. A total of 164 patients were included in the study. Preoperative assessments included chest computed tomography (CT), abdominal CT, brain magnetic resonance imaging (MRI) and bone scanning with neck sonography. Since 2004, the routine preoperative assessment has included chest CT, positron emission tomography (PET)-CT, bone scanning, brain MRI, and bronchoscopy. Thirty-one patients underwent sublobar resection, and 133 patients underwent lobectomy. Sublobar resection was considered for severe underlying lung disease (interstitial lung disease, obstructive lung disease), poor cardiac function (valvular heart disease, coronary disease), aortic dissection or aneurysm and other underlying malignancy. Of patients undergoing sublobar resection, 12 patients underwent segmentectomy, and 19 patients underwent wedge resection with or without lymph node dissection. Pathologic features and clinical outcomes were compared between the two patient groups. Recurrence was defined as local or extrathoracic metastasis and was based on clinical and pathological evidence. Patients with pathologic stage II or III received adjuvant chemotherapy or chemoradiotherapy. The patients with sublobar resection received radiotherapy if the resected margin was positive.

### Statistical analysis

All statistical analyses were carried out using SPSS version 18 (SPSS Inc., Chicago, USA). Continuous variables were compared using the Mann-Whitney *U* test, and categorical variables were compared using the Chi-square test and the Fisher exact test. Disease-free intervals and overall survival rates were estimated using the Kaplan-Meier method and compared using the log-rank test. Variables associated with recurrence and survival were determined using the Cox proportional hazards model. Before application of the Cox proportional hazards model, the proportionality assumption was checked. Multivariate analysis for disease-free interval and survival was also performed using the Cox proportional hazards model. Variables with a *P* value less than 0.05 by univariate analysis were finally evaluated by multivariate analysis using forward selection.

## Results

Records of a total of 164 patients were reviewed in this study. High-risk patients tended to undergo sublobar resection (Table 1). Sublobar resection was significantly associated with older age, more cardiovascular disease (*P* = 0.001), and low diffusing capacity of the lung for carbon monoxide (DLCO). The mean age of lobectomy patients and sublobar resection patients was 62.53 and 69.65 years, respectively (*P* <0.001). Eight patients (6%) with cardiovascular disease underwent lobectomy, and nine patients (29%) underwent sublobar resection. DLCO was significantly lower in the sublobar resection patients (*P* <0.001).

**Table 1:** Baseline patient characteristics

**Characteristic**	**Lobectomy (N = 133)**	**Sublobar resection (N = 31)**	***P *** **value**
	**Mean ± SD or n (%)**	**Mean ± SD or n (%)**	
Age	62.53 ± 9.12	69.65 ± 10.94	<0.001
Sex male	83 (62.41)	22 (70.97)	0.413
Hypertension	49 (36.84)	14 (45.16)	0.417
Diabetes mellitus	19 (14.29)	8 (25.81)	0.175
Cardiovascular disease	8 (6.02)	9 (29.03)	0.001
Cerebrovascular accident	3 (2.26)	3 (9.68)	0.082
Interstitial lung disease	11 (8.27)	11 (19.35)	0.096
COPD	6 (4.51)	4 (12.90)	0.096
FEV1	2.41 ± 0.57	2.16 ± 0.66	0.087
DLCO	98 ± 19.78	75 ± 15.88	<0.001
PET SUVmax	4.81 ± 3.41	5.85 ± 4.45	0.386

Data presented as the mean (standard deviation) or frequencies and percentages as appropriate.

COPD, chronic obstructive pulmonary disease; DCLO, diffusing capacity of the lung for carbon monoxide; FEV1, forced expiratory volume-one second; PET SUVmax, maximum standardized uptake value on positron emission tomography. Data presented as mean ± SD or n (%) as appropriate. Continuous variables were compared using Mann-Whitney *U* test and categorical variables were tested using Chi-square test or Fisher exact test.

Pathologic variables are listed in Table 2. Adenocarcinoma was the most common tumor cell type; however, the fraction of adenocarcinomas was significantly higher in the lobectomy patients (*P* = 0.012). There was no significant difference in the degree of tumor differentiation between the two groups. The mean tumor size of the lobectomy and sublobar resection patients was 2.19 cm and 1.99 cm, respectively (*P* = 0.239). Among lobectomy patients, the mean bronchial margin was 3.51, and among sublobar resection patients, the mean margin was 0.95 cm (*P* <0.001). The number of excised lymph nodes was significantly higher in the lobectomy group (*P* <0.001). Twenty patients with lobectomy (15%) had pathologic nodal disease (*P* <0.015). The number of positive lymph nodes was significantly higher in the lobectomy patients (*P* = 0.019). Although the number of metastatic mediastinal lymph nodes was higher in the lobectomy patients, the difference was not significant. There was no significant difference in the numbers of patients with lymphatic vessel invasion, visceral pleural invasion, and blood vessel invasion between the two groups.

**Table 2:** Pathologic data of solid-type, clinical stage IA, non-small cell lung cancers

**Variables**	**Lobectomy (N = 133)**	**Sublobar resection (N = 31)**	***P *** **value**
	**Mean ± SD or n (%)**	**Mean ± SD or n (%)**	
**Histology**			0.012
**Adenocarcinoma**	109 (82.0)	18 (58.1)	
**Squamous**	16 (12.0)	10 (32.36)	
**Others**	8 (6.0)	3 (9.7)	
**Differentiation**			0.116
**Well**	51 (38.4)	6 (19.4)	
**Moderate**	65 (48.9)	21 (67.7)	
**Poor**	17 (12.8)	4 (12.9)	
**Tumor size**	2.19 ± 0.75	1.99 ± 0.75	0.239
**p T stage**			0.694
**pT1a**	67 (50.4)	17 (54.8)	
**Margin**	3.51 ± 2	0.95 ± 1.16	<0.001
**Positive margin**	1 (0.8)	7 (22.6)	<0.001
**Visceral pleural invasion**	15 (11.3)	3 (6.7)	1.000
**Lymphatic vessel invasion**	29 (21.8)	4 (12.9)	0.450
**Blood vessel invasion**	3 (2.3)	0 (0)	1.000
**Number of LN harvest**	10.50 ± 7.6	1.74 ± 5.09	<0.001
**LN metastasis**	20 (15.0)	0 (0)	0.015
**Number of positive LNs**	0.53 ± 1.90	0 (0)	0.019
**N2 positive**	10 (7.5)	0 (0)	0.211
**p Stage**			0.156
**p Stage I**	112 (84.2)	30 (96.8)	
**p Stage II**	11 (8.3)	0 (0)	
**p Stage III**	10 (7.5)	1 (3.2)	
**Recurrence**	35 (26.3)	19 (61.3)	<0.001
**Lung to lung metastasis**	8 (6)	6 (19.4)	
**Margin**	4 (3)	4 (12.9)	
**Mediastinal lymph node**	6 (4.5)	5 (16.1)	
**Distant metastasis**	17 (12.8)	4 (12.9)	

Data presented as the mean (standard deviation) or frequencies and percentages as appropriate.

LN, lymph nodes. Data presented as mean ± SD or n (%) as appropriate. Continuous variables were compared using Mann-Whitney *U* test, and categorical variables were tested by Chi-square test or Fisher exact test.

**Figure 1:**
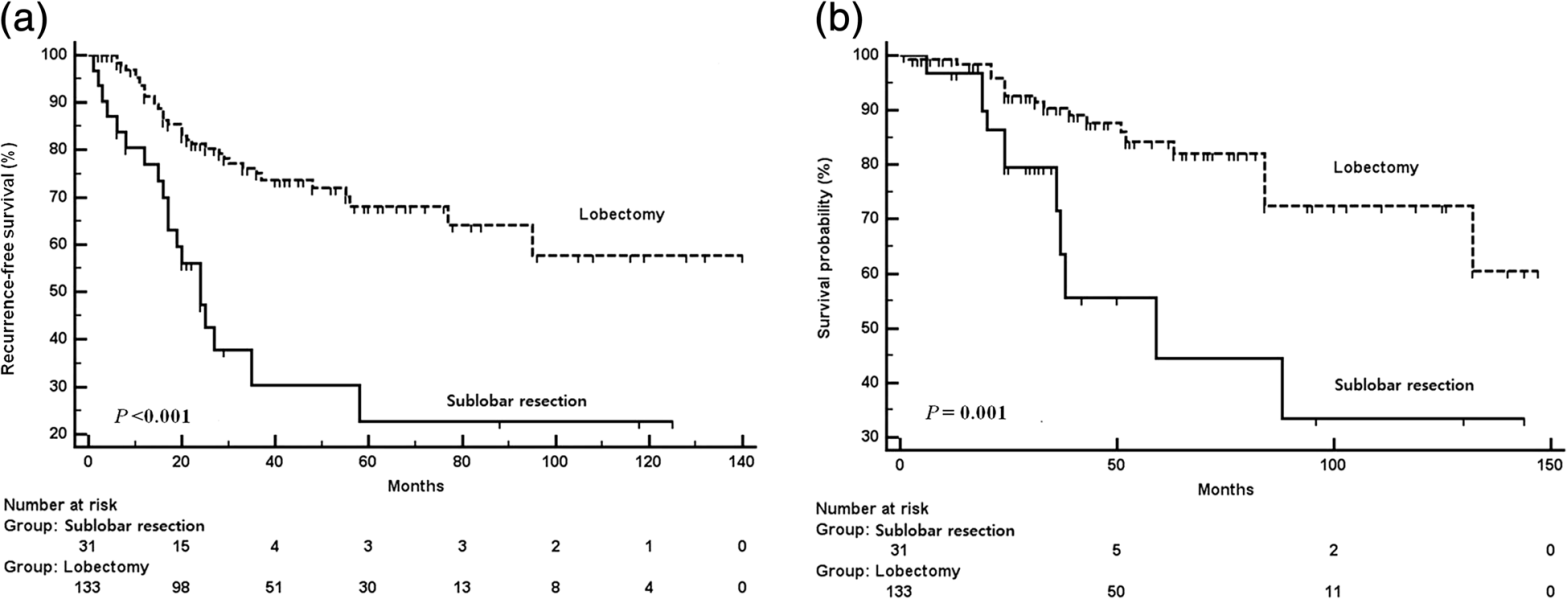
**Survival and disease-free intervals of patients undergoing lobectomy and sublobar resection for solid-type, clinical stage IA, non-small cell lung cancers. a**. Recurrence-free survival. **b**. Overall survival.

All patients were followed until recurrence and death or loss to follow-up (F/U). The median F/U period for lobectomy and sublobar resection patients was 40 months and 30 months, respectively. There was recurrence in 35 lobectomy patients (26%) and 19 sublobar resection patients (61%; *P* <0.001). Local recurrence rate was also significant higher in the patients with sublobar resection (7.5% in lobectomy versus 29% in sublobar resection; *P* <0.001). The disease-free interval of lobectomy patients was significantly longer (*P* <0.001; Figure 1a). By univariate analysis, cardiovascular disease, sublobar resection, visceral pleural invasion, positive margin, lymphatic vessel invasion, and lymph node metastasis were significant predictors of recurrence. By multivariate analysis, sublobar resection (*P* <0.001), visceral pleural invasion (*P* = 0.002), and lymphatic vessel invasion (<0.001) were significant for recurrence (Table 3).

**Table 3:** Univariate and multivariate analyses for predictors of recurrence

**Variables**	**Univariate**			**Multivariate**		
	**HR**	**95% CI**	***P value***	**HR**	**95% CI**	***P value***
**Cardiovascular disease**	2.152	1.051 to 4.409	0.036			
**Sublobar resection**	3.379	1.920 to 5.946	<0.001	4.387	2.438 to 7.891	<0.001
**Visceral pleural invasion**	3.281	1.628 to 6.612	0.001	3.142	1.510 to 6.538	0.002
**Positive margin**	2.692	1.148 to 6.314	0.023			
**Lymphatic vessel invasion**	3.308	1.853 to 5.908	<0.001	3.385	1.853 to 6.182	<0.001
**Lymph node metastasis**	2.194	1.067 to 4.512	0.033			

CI, confidence interval; HR, hazard ratio.

Sublobar resection was associated with high overall mortality. (*P* = 0.001; Figure 1b). Estimated 5-year survival rate was 81% in the lobectomy group and 41% in the sublobar resection group. Univariate analysis for predictors of survival revealed that age, cardiovascular disease, sublobar resection, lymphatic vessel invasion, number of lymph node metastases, and pathological mediastinal lymph node metastasis were statistically significant. Sublobar resection (*P* = 0.011), lymphatic vessel invasion (*P* = 0.006), and number of metastatic lymph nodes (*P* = 0.028) were significant predictors for overall survival by multivariate analysis (Table 4). Only sublobar resection and lymphatic vessel invasion were significant for both recurrence and survival in our study.

**Table 4:** Univariate and multivariate analyses for predictors of survival

**Variables**	**Univariate**			**Multivariate**		
	**HR**	**95% CI**	***P value***	**HR**	**95% CI**	***P value***
**Age**	1.050	1.010 to 1.091	0.014			
**Cardiovascular disease**	3.501	1.550 to 7.906	0.003			
**Sublobar resection**	3.236	1.525 to 6.868	0.002	3.224	1.312 to 7.924	0.011
**Lymphatic vessel invasion**	3.690	1.670 to 8.154	0.001	3.586	1.445 to 8.900	0.006
**Number of positive LNs**	1.272	1.095 to 1.478	0.002	1.214	1.022 to 1.442	0.028
**N2 positive**	5.123	1.716 to 15.297	0.003			

CI, confidence interval; HR, hazard ratio; LN, lymph node; N2, mediastinal lymph node.

**Figure 2:**
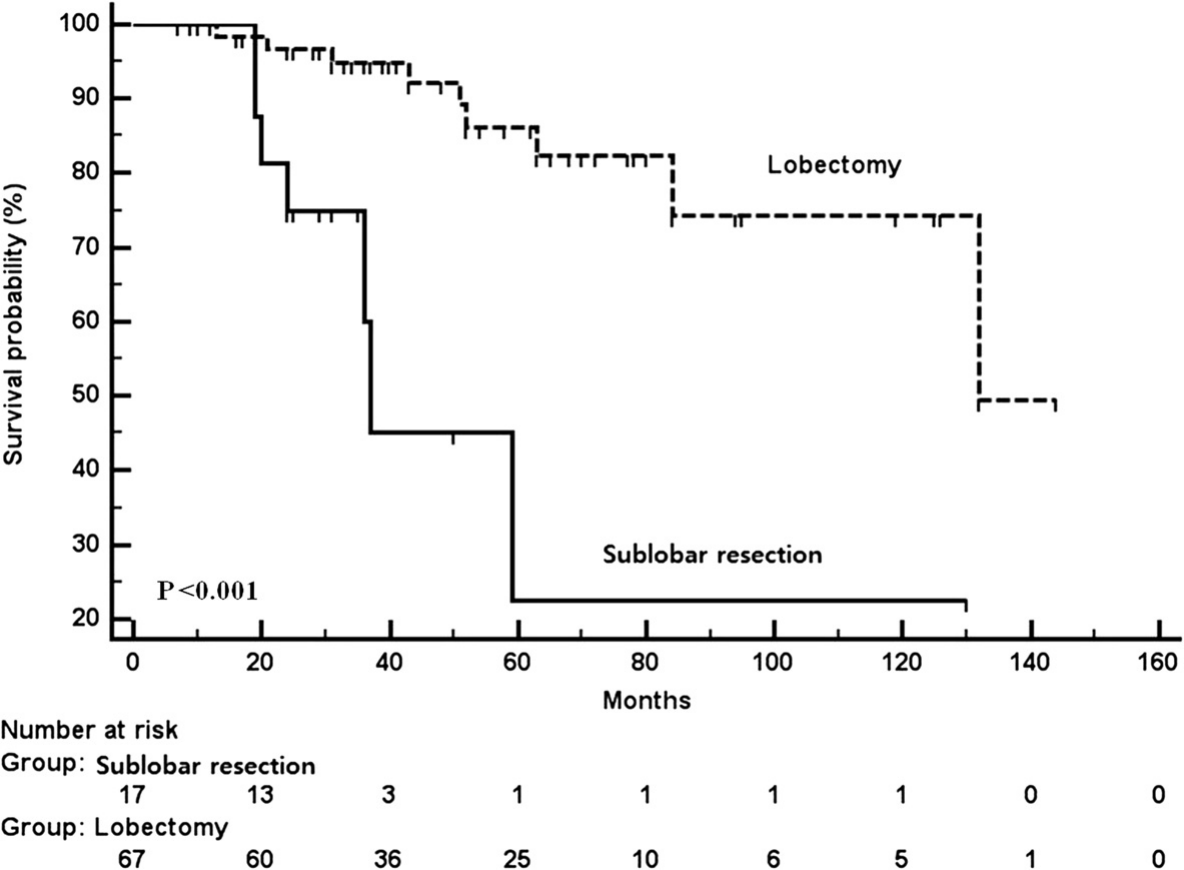
Overall Survival patients undergoing lobectomy and sublobar resection for solid-type, clinical stage IA, non-small cell lung cancers smaller than 2 cm.

In a subgroup analysis of 83 patients with tumors smaller than 2 cm, lobectomy was significantly associated with better clinical outcomes than sublobar resection (Figure 2). We also evaluated clinical outcomes between segmentectomy and wedge resection in 31 patients with sublobar resections. There was no significant difference for disease-free interval (*P* = 0.252) and survival (*P* = 0.821) between segmentectomy and wedge resection.

There was no significant difference for survival (*P* = 0.979) and disease-free interval (*P* = 0.707) according to tumor size (pT1a versus pT1b) in sublobar resection.

Multivariate analysis after exclusion of the eight patients with incomplete resection demonstrated that sublobar resection was the only independent risk factor for overall survival in subgroup analysis (*P* = 0.038, Hazard ratio: 3.01, 95% confidence interval, 1.06 to 8.58).

## Discussion

Lung cancer is one of the most common malignant tumors worldwide, with a poor prognosis despite multimodality management [5]. However, 5-year survival rates of up to 70% have been achieved after surgical treatment for patients with stage I lung cancers [6]. Pneumonectomy, with high morbidity and mortality, was the standard surgical treatment in the 1940s [7]. Since the 1950s, lobar resection plus mediastinal lymph node dissection has become the standard surgical treatment for lung cancer [8,9].

Patients with early lung cancer who are not suitable for lobar resections are a dilemma for thoracic surgeons. Most of these patients are elderly and have poor cardiopulmonary reserve and other medical comorbidities. Sublobar resection has been an alternative treatment, and its efficacy has been evaluated in several studies [10,11]. Ground-glass opacities (GGO) or GGO-predominant lesions on chest CT are features of bronchioloalveolar carcinoma or adenocarcinoma, which are slowly growing and noninvasive tumors [12]. Sublobar resection is appropriate for these lesions [13-15]; however, the efficacy of sublobar resection for solid-type lung cancer is controversial.

In 1995, a randomized trial was performed that compared sublobar resection with lobectomy for clinical early stage NSCLC [2]. Sublobar resection was associated with high rates of recurrence and death. Landreneau *et al*. compared wedge resection with lobectomy in 1997 [3]. Wedge resection failed to demonstrate equivalent results; however, Kodama *et al*. demonstrated that sublobar resection with lymph node dissection was not inferior to lobectomy [16]. Because of advances in imaging study, surgical technique and revised TNM classification, the effect of sublobar resection has been evaluated for patients with clinical T1a lung cancers again. Several studies found no difference for recurrence and survival between patients undergoing sublobar resection or lobectomy [4,17,18]. Okada *et al*. compared segmentectomy with lobectomy for tumors smaller than 2 cm, and found respective 5-year survival rates of 87.1% and 87.7% for segmentectomy and lobectomy. In recent study, randomized study was conducted and sublobar resection is not inferior to lobectomy for clinical stage IA lung cancer [19]. The recurrence rate was more common in the patients with sublobar resection but not significantly and the survival was not different in their study.

Our study showed different results. High-risk patients more frequently underwent sublobar resection, and sublobar resection showed shorter disease-free interval and poor survival. Furthermore, 19 patients (61.3%) in sublobar resection showed recurrence during the follow-up period. Lung to lung metastasis (31.6%) was most common followed by mediastinal lymph node metastasis (26.3%).

Lymph node metastasis is common in solid-type lung cancers [20]. In our study, 15% of lobectomy patients had lymph node metastases at the time of surgery in clinical stage IA NSCLC. Lymphatic vessel invasion has been found to be a prognostic factor [21]. Lymphatic vessel invasion is associated with lymph node metastasis, recurrence, and decreased survival. Funai *et al*. reported that lymphatic vessel invasion was an independent prognostic factor for early-stage lung cancers [22].

The proportion of solid component has been correlated with malignant potential regardless of tumor size [23]. Our study results support this finding. Despite the early stages of the solid-type tumors in our study, they had aggressive behavior and were associated with several prognostic pathologic features, including lymphatic vessel invasion, pleural invasion, and occult lymph node metastasis. In our study, sublobar resection failed to demonstrate efficacy, even for tumors smaller than 2 cm. Lobectomy provided longer disease-free-interval and survival. Therefore, lobectomy with lymph node dissection remains standard treatment for patients with solid-type early lung cancers, regardless of tumor size.

Our study had limitations because it was small and non-randomized, and positive margins were common among the sublobar resections; however, neither recurrence nor survival was associated with positive margins according to multivariate analysis. Okami *et al*. found that sublobar resection for elderly patients provided good clinical outcomes [24], but we did not compare sublobar resection with lobectomy for high-risk patients, including patients who were older or who had poor cardiopulmonary reserve. Another limitation was that the number of resected lymph nodes evaluated from patients undergoing sublobar resection was significantly lower than the number from lobectomy patients [25,26].

## Conclusions

In conclusion, solid-type lung cancers demonstrated aggressive behavior, and there were numerous significant pathologic prognostic factors in clinical stage IA NSCLC from our study. Lymph node metastasis was common in clinical stage IA NSCLC with a solid component. Lobectomy with lymph node dissection remains the standard surgical procedure for patients with solid-type, clinical stage, IA NSCLC.

## Abbreviations

CT: computed tomography; DLCO: diffusing capacity of the lung for carbon monoxide; F/U: follow-up; GGO: ground-glass opacity; MRI: magnetic resonance imaging; NSCLC: non-small cell lung cancer; PET: positron emission tomography.

## Competing interests

The authors declare that they have no conflicts of interest or financial ties to disclose.

## Author’s contributions

HWJ carried out the review of medical records, analysis and writing. YDK and SWS carried out the revision. KSK carried out the review of medical records. HJP carried out the revision of statistics. JKP carried out the correspondence, revision, and review of medical records. All authors read and approved the final manuscript.
